# A case of groove pancreatitis with duodenal stenosis successfully treated by endoscopic ultrasonography‐guided pancreaticogastrostomy

**DOI:** 10.1002/deo2.190

**Published:** 2022-11-30

**Authors:** Muneo Ikemura, Ko Tomishima, Hiroto Ota, Daishi Kabemura, Mako Ushio, Taito Fukuma, Sho Takahashi, Akinori Suzuki, Yusuke Takasaki, Koichi Ito, Shigeto Ishii, Toshio Fujisawa, Hiroyuki Isayama

**Affiliations:** ^1^ Department of Gastroenterology, Graduate School of Medicine Juntendo University Tokyo Japan

**Keywords:** duodenal stenosis, EUS‐PD, EUS‐PGS, groove pancreatitis, Santorini's duct

## Abstract

One of the reasons for groove pancreatitis is caused by the leakage of pancreatic juice into the space between the pancreatic head, descending duodenum, and common bile duct. Endoscopic drainage of Santorini's duct (SD) via the minor papilla is reportedly efficacious but can be difficult due to duodenal stenosis. We report Santorini's duct drainage using endoscopic ultrasonography‐guided pancreaticogastrostomy (EUS‐PGS) for a case of groove pancreatitis with gastric outlet obstruction. Gastric outlet obstruction was improved after 7 months of EUS‐PGS with internal drainage through the Santorini's duct/minor papilla. EUS‐PGS may be effective for treating groove pancreatitis with duodenal stenosis. This is the first report of groove pancreatitis with duodenal stenosis, the symptoms of which were improved by EUS‐PGS.

## INTRODUCTION

Groove pancreatitis is a form of chronic pancreatitis, in which inflammation affects the groove area confined by the pancreatic head, descending duodenum, and common bile duct. The disease is uncommon but accounts for 12.8%–19.5% of pancreatoduodenectomies performed for chronic pancreatitis due to failure of conservative treatment or misdiagnosis as pancreatic cancer.^1^ Stopping the leakage of pancreatic juice by draining Santorini's duct (SD) via the minor papilla is an effective endoscopic treatment modality but can be difficult in cases of duodenal stenosis.^2^ We report a case of groove pancreatitis in which duodenal stenosis was improved by endoscopic ultrasonography‐guided drainage of the pancreatic duct, and a good clinical course was achieved.

## CASE REPORT

**FIGURE 1:**
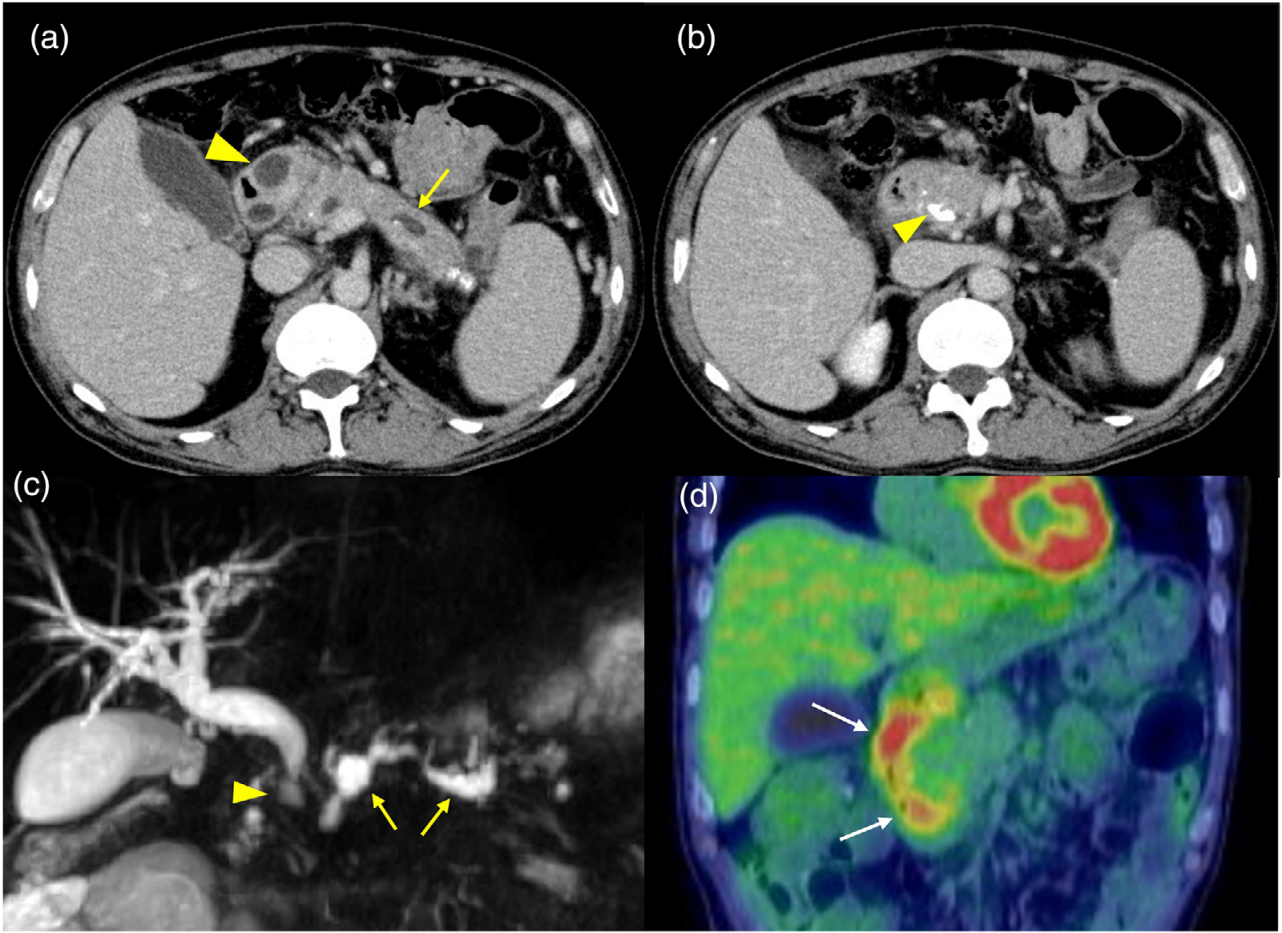
(a) Computed tomography: Duodenal wall thickening and cysts are observed (arrowhead). The main pancreatic duct is dilated (arrow). (b) Computed tomography: The pancreatic head is enlarged, and the surrounding fatty tissue density is elevated. A pancreatic stone is seen in the pancreatic head (arrowhead). (c) Magnetic resonance cholangiopancreatography: The distal bile duct is gently and smoothly stenosed (arrowhead), and the bile duct is dilated. The main pancreatic duct also shows stenosis at the head and cyst‐like dilatation toward the body's tail (arrow). (d) Fluorine‐18‐deoxyglucose: Fluorine‐18‐deoxyglucose was accumulated from the upper part of the duodenum to the inferior duodenal angle (arrow). The accumulation was along the wall of the duodenum and was considered due to inflammation in the groove area

A 55‐year‐old male with a history of chronic alcoholic pancreatitis with pancreatic calculi. He had consumed a bottle of wine daily (90 g pure ethanol/day) for 20 years and was treated medically and endoscopically for 8 years. He had suffered from abdominal pain and recurrent acute exacerbation of chronic pancreatitis 2 years before coming to our hospital. Contrast‐enhanced computed tomography (CT) revealed pancreatic stones, dilatation of the main pancreatic duct, thickening of the duodenal wall, cystic lesions, and inflammation in the groove area. Esophagogastroduodenoscopy revealed duodenal stenosis without tumor involvement. A pancreatic plastic stent was inserted for stenosis in a different hospital, and the stent was replaced periodically. Because of duodenal stenosis, the stent was removed using the forward‐viewing scope 5 years before visiting our hospital. After that, he was treated medically. In March 2020, he was referred to our hospital for treatment of groove pancreatitis with gastric outlet obstruction. At admission, his abdomen was flat, soft, and mildly painful in the upper area. Laboratory findings on admission showed a high CA19‐9 level of 75 U/L (Table 1). Contrast‐enhanced CT of the abdomen showed a mildly enlarged pancreatic head with an increased CT value in fat in the surrounding area. A cystic lesion in the groove area and duodenal wall thickening were observed with dilation of the main pancreatic duct and numerous pancreatic stones (Figure 1a,b). Magnetic resonance cholangiopancreatography showed that the distal bile duct was gently and smoothly narrowed, and the bile duct was dilated (Figure 1c). He was recommended both surgical treatment and interventional treatment because of the impossibility of endoscopic transpapillary stent insertion, and he chose interventional treatment, that is to say, endoscopic ultrasonography‐guided pancreaticogastrostomy (EUS‐PGS) with drainage of SD as more minimally invasive treatment. Then we performed positron emission tomography (PET)‐CT. It showed fluorine‐18‐deoxyglucose accumulation from the upper duodenum to the inferior duodenal angle (Figure 1d).

**TABLE 1:** Admission blood tests

CBC		Biochemistry			
WBC	4400 /µl	TP	6.9 g/dl	Cl	107 mEq/l
RBC	472×104/µl	Alb	3.9 g/l	Ca	9.2 mg/dl
Hb	14.3g /dl	T‐Bil	1.10 mg/dl	Amy	127 U/l
Hct	41.2%	D‐Bil	0.05 mg/dl	Lypase	142 U/l
MCV	87.3 fl	AST	26 U/l	CRP	0.07 mg/dl
MCH	30.3 pg	ALT	21 U/l	HbA1c (NGSP)	5.3%
MCHC	34.7 g/dl	LDH	173 U/l	**Etc**.	
Plt	20.5×104 /µl	ALP	265 U/l	IgG	1051 mg/dl
**Coagulation**		γ‐GTP	49 U/l	IgA	324 mg/dl
PT	97.2 µ/l	BUN	11 mg/d	IgM	76 mg/dl
PT‐INR	1.04	Cre	0.53 mg/dl	CA19‐9	75 U/l
APTT	31.8 s	Na	144 mEq/l	CEA	2.4 ng/ml
APTT‐CO	31.8 s	K	4.1 mEq/l	Dupan 2	<25 U/ml
				Span‐1	25 U/ml

γ‐GTP, γ‐glutamyl transpeptidase; Alb, albumin; ALP, alkali‐phosphatase; ALT, alanine aminotransferase; AMY, amylase; APTT, activated partial thromboplastin time; AST, aspartate aminotransferase; BUN, blood urea nitrogen; Ca, calcium; CA19‐9, carbohydrate antigen 19‐9; CEA, carcinoembryonic antigen; Cl, chlorine; Cr, creatinine; CRP, C‐reactive protein; D‐Bil, direct bilirubin; DUPAN‐2, duke pancreatic monoclonal antigen type 2; Hb, hemoglobin; HbA1c (NGSP), HbA1c (National Glycohemoglobin Standardization Program); Hct, hematocrit; K, kalium; LDH, lactate dehydrogenase; MCH, mean corpuscular hemoglobin; MCHC, mean corpuscular hemoglobin concentration; MCV, mean corpuscular hemoglobin; Na, natrium; PLT, platelet; PT, prothrombin time, PT‐INR, PT‐international normalized ratio; RBC, red blood cell; Span‐1, s‐pancreas‐1 antigen; T‐Bil, total bilirubin; TP, total protein; WBC, white blood cell.

**FIGURE 2:**
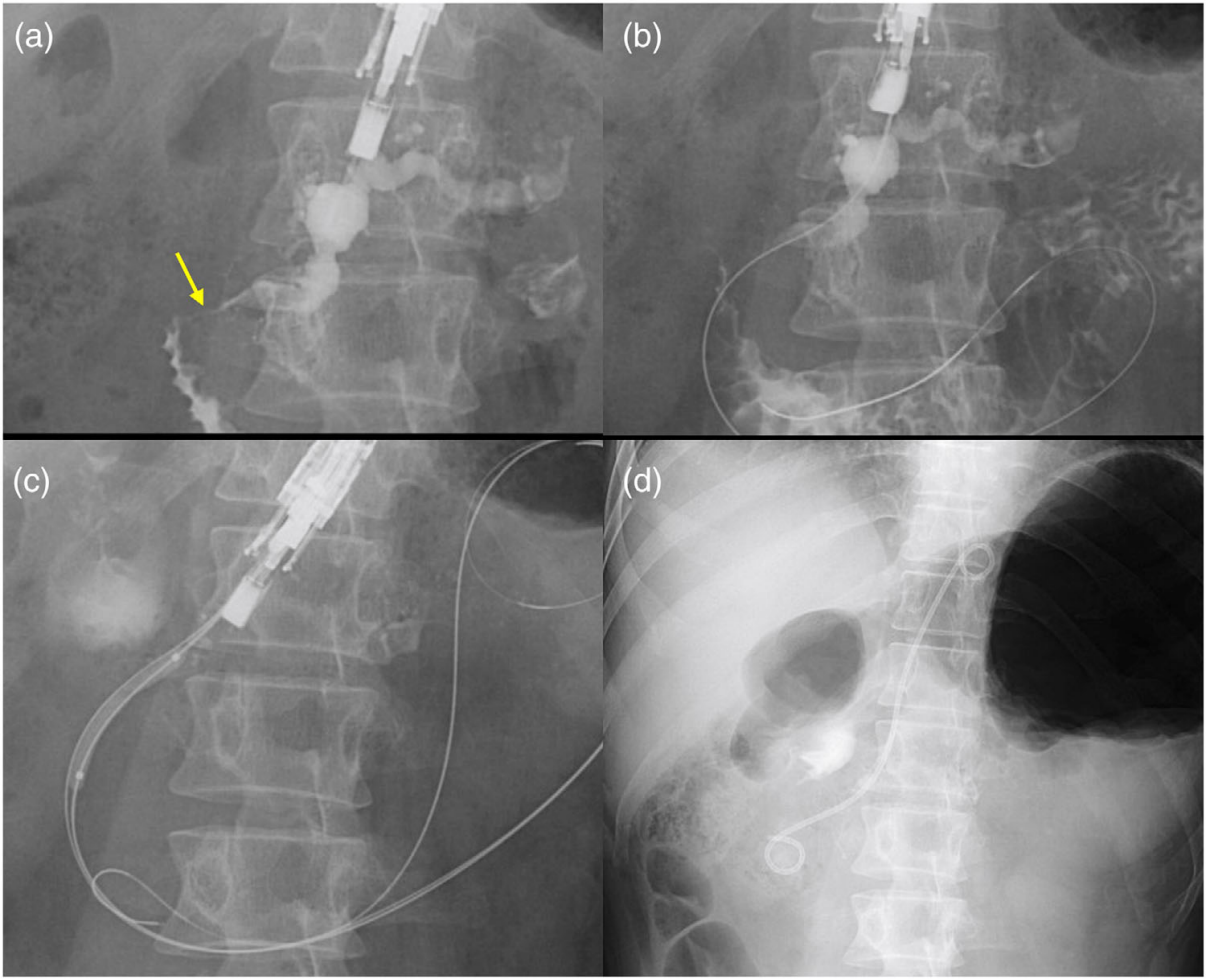
Endoscopic ultrasonography‐guided pancreaticogastrostomy: (a) The main pancreatic duct was punctured from the pancreatic body with a 19‐gauge needle (EZshot3; Olympus Medical Systems, Tokyo, Japan). Pancreatogram showed both the stenosis of Santorini's (arrow) and Wilsung's ducts. (b) A 0.025‐inch guidewire (Visiglide2; Olympus Medical Systems) was successfully passed through the Santorini's duct and minor papilla and placed in the duodenum. (c) Double guidewire technique with additional 0.035‐inch hard type guidewire placement (RevoWave, Ultrahard type; Piolax Medical Devices Inc., Kanagawa, Japan) was feasible and safe to dilate by balloon (REN; KANEKA Medical, Osaka, Japan). (d) A 7‐Fr x 15‐cm pigtail stent (Zimmon; Cook Medical, Bloomington, IN, USA) was placed through the Santorini's duct stenosis and minor papilla via the Endoscopic ultrasonography‐guided pancreaticogastrostomy route

**FIGURE 3:**
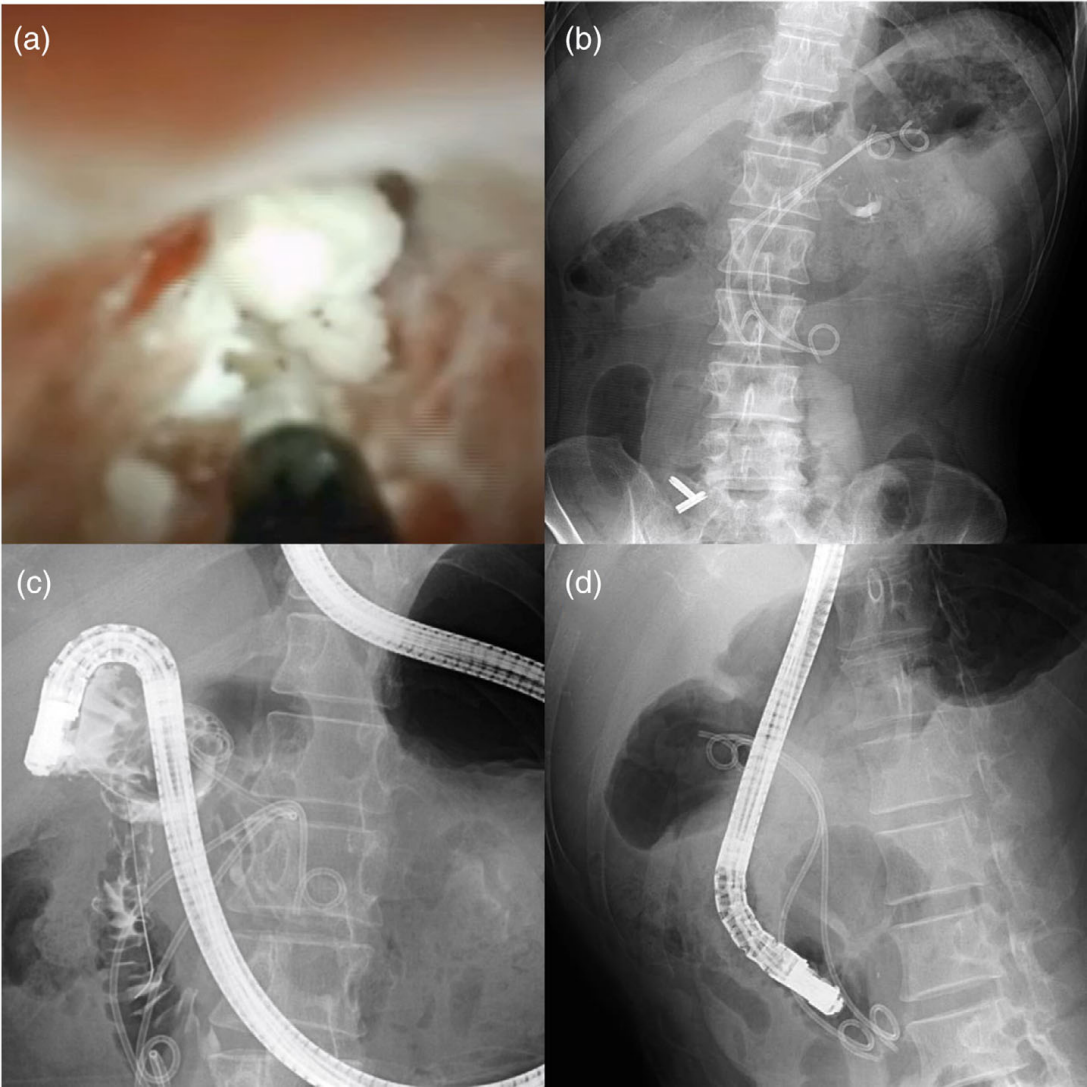
Electrohydraulic lithotripsy with pancreatoscope from endoscopic ultrasonography‐guided pancreaticogastrostomy (route and duodenoscope passage. (a) Electrohydraulic lithotripsy under a pancreatoscope (SpyScope DS; Boston Scientific, Marlborough, MA, USA) was performed for pancreatic stone treatment after dilation of the EUS‐PGS anastomosis. (b) After the pancreatic stone was crushed completely, two 7‐Fr × 12‐cm pigtail stents were placed in each Wirsung's and Santorini's duct. (c) At 1 month after the EUS‐PGS, the duodenal stricture was difficult to pass the duodenal stenosis. (d) At 7 months after the endoscopic ultrasonography‐guided pancreaticogastrostomy, a duodenoscope could pass the duodenal stenosis

EUS‐PGS was performed using a convex‐type echoendoscope (EG‐580UT; Fujifilm Corp., Tokyo, Japan) by puncturing the main pancreatic duct with a 19‐gauge needle (EZshot3; Olympus, Tokyo, Japan) following contrast injection (Figure 2a) and insertion of a 0.025‐inch guidewire (Visiglide2; Olympus) (Figure 2b). Pancreatography showed stenosis of SD (Figure 2a, arrow) and Wirsung's duct. A guidewire was used to pass SD and minor papilla and was placed in the duodenum. The double‐guidewire technique with the placement of an additional 0.035‐inch hard‐type guidewire (RevoWave, Ultrahard type; Piolax Medical Devices Inc., Kanagawa, Japan) was employed to insert a plastic stent to keep the alignment straight during device insertion and promote endoscopic stability.^3^ Furthermore, by leaving additional safety guidewire, even after failed stent insertion over the initial guidewire, EUS‐guided drainage can be achieved by the second guidewire. We dilated the puncture tract using a boogie dilator (ES Dilator; Zeon Medical devices, Kanagawa, Japan), 6.5‐Fr cautery dilator (Cysto‐gastro‐set; ENDO‑FLEX, Voerde, Germany), and 4‐mm balloon catheter (Ren; Kaneka Medical, Osaka, Japan; Figure 2c). We usually use a boogie dilator followed by a balloon catheter. But in case of very stiffness of the pancreas duct, we used a cautery dilator to pass the pancreatic wall. A 7‐Fr × 15‐cm pig‐tail stent (Zimmon; Cook Medical, Bloomington, IN) was passed through the SD stenosis and minor papilla (Figure 2d). Numerous pancreatic stones were found as filling defects in the main pancreatic duct. Pancreatic leakage requiring temporary EUS‐guided drainage occurred after the procedure. One month later, in the second session, electrohydraulic lithotripsy under a pancreatoscope (SpyScope DS; Boston Scientific, Marlborough, MA) was performed to treat the pancreatic stones after additional 4 mm balloon dilation of the EUS‐PGS anastomosis from the fistula site (Figure 3a), and two 7‐Fr × 12‐cm pig‐tail stents were placed in Wirsung's and SDs (Figure 3b). Thereafter, the plastic stents were exchanged every 3 months. Although, at one month after the EUS‐PGS, a duodenoscope was difficult to pass the duodenal stenosis (Figure 3c), at 7 months after the initial session, could be passed through the duodenal stenosis (Figure 3d), and stent exchange through the minor and major papilla was possible. And after that, we did stent exchange through the papilla periodically. Inflammation in the groove area and around the duodenum improved after the drainage. Abdominal pain was alleviated, and duodenal stenosis improved gradually. The patient has been symptom‐free for 2 years since the stenosis was resolved, and the stents in Wirsung's and SDs remain in situ.

## DISCUSSION

Differential diagnosis of pancreatic cancer is important but difficult in groove pancreatitis.^4^ In this case, the pattern of fluorine‐18‐deoxyglucose uptake and the cystic lesions in the groove area with involvement of the duodenal wall pointed towards the diagnosis of groove pancreatitis. Additionally, PET‐CT was performed twice in 1 year, and no finding indicative of tumor development was observed. Because there was no tumor lesion in other modalities, such as enhanced CT and EUS, EUS‐FNA had no indication. Duodenal stenosis due to malignancy mainly has bile duct obstruction, but this case had no obstruction, such as elevation of serum bilirubin, with only smooth bile duct stricture. Based on these results, the possibility of malignancy was considered infinitesimally low. When we performed EUS‐PGS, we confirmed pancreas juice cytology was no malignancy. However, since features of pancreatic cancer and groove pancreatitis frequently overlap, we need to exercise a high degree of caution while interpreting the images.^5^, ^6^ Because of its rarity, there is a paucity of data on the 18F‐fluorine‐18‐deoxyglucose PET/CT features of groove pancreatitis.

The mechanism of groove pancreatitis is thought to be induction by long‐term alcohol intake of hyperplasia of Brunner's glands and secretion of highly viscous pancreatic juice, resulting in an impaired outflow of pancreatic juice, which leaks into the groove area.^7^ Nonaccessible or nontraversable papillae/surgical anastomoses are the main indications for EUS‐PGS. The tips to succeed in EUS‐PGS for chronic pancreatitis are puncture and guidewire seeking. Since the diameter of the pancreatic duct is thin and the parenchyma is hard due to recurrent pancreatitis, we try to speed up the puncture and use the Selzinger method to penetrate the pancreatic duct and then bring the needle back to align the tip with the pancreatic duct. To insert the stents or some devices, we dilate the fistula with a 4‐mm balloon device. To prevent pancreatic juice leakage, dilating the cause stricture and releasing the pancreatic juice is also important. In the second session, pancreatoscopy through the matured fistula created by EUS‐PGS is feasible and effective and can diagnose and treat with acceptable adverse event rates.^8^ The advantage of this technic is that transgastric or transenteric drainage is preferable in terms of patient comfort and decreased risk of percutaneous chronic fistula formation.

Most cases of groove pancreatitis with duodenal stenosis undergo surgical management because of the difficulty of differential diagnosis of pancreatic cancer and the impossibility of endoscopic passage of the papilla.^9^ However, diagnostic imaging and endoscopic modalities have advanced the treatment of groove pancreatitis. In particular, interventional EUS has undergone remarkable progress in recent years, and EUS‐guided fine needle aspiration is typically used to treat pancreaticobiliary disease.^10^ The effects on pancreatic endocrine and exocrine functions and the risk of cancer development due to the long‐term maintenance of pancreaticogastric fistula are also unknown. We think that duodenal stenosis due to groove pancreatitis can be resolved by drainage of Santorini's and Wirsung's ducts like in this case. If the duodenoscope can be passed up to the duodenal papilla, we replace the pancreatic stent via minor and major papilla and want to close the pancreaticogastric fistula in the future. We report a case of groove pancreatitis with duodenal stenosis, the symptoms of which were improved by EUS‐PGS.

## CONFLICT OF INTEREST

Isayama H is supported by honoraria from the Fujifilm Corp., Tokyo, Japan.

## References

[1] Adsay NV , Zamboni G . Paraduodenal pancreatitis: A clinico‐pathologically distinct entity unifying “cystic dystrophy of heterotopic pancreas”, “para‐duodenal wall cyst”, and “groove pancreatitis”. Semin Diagn Pathol 2004; 21: 247–54. 16273943 10.1053/j.semdp.2005.07.005

[2] Isayama H , Kawabe T , Komatsu Y *et al*. Successful treatment for groove pancreatitis by endoscopic drainage via the minor papilla. Gastrointest Endosc 2005; 61: 175–8. 15672084 10.1016/s0016-5107(04)02460-5

[3] Nakai Y , Oyama H , Kanai S *et al*. Double guidewire technique using an uneven double lumen catheter for endoscopic ultrasound‐guided interventions. Dig Dis Sci 2021; 66: 1540–7. 32436121 10.1007/s10620-020-06345-9

[4] Yamaguchi K , Tanaka M . Groove pancreatitis masquerading as pancreatic carcinoma. Am J Surg 1992; 163: 312–6. 1539765 10.1016/0002-9610(92)90009-g

[5] Levenick JM , Gordon SR , Sutton JE , Suriawinata A , Gardner TB . A comprehensive, case‐based review of groove pancreatitis. Pancreas 2009; 38: e169–75. 19629001 10.1097/MPA.0b013e3181ac73f1

[6] Parihar AS , Singh H , Kumar R , Gupta V , Singh H , Mittal BR . Pancreatic malignancy or not?: Role of 18F‐FDG PET/CT in solving the diagnostic dilemma and evaluating treatment response. Clin Nucl Med 2018; 43: e115–7. 29401142 10.1097/RLU.0000000000001989

[7] Chantarojanasiri T , Isayama H , Nakai Y *et al*. Groove pancreatitis: Endoscopic treatment via the minor papilla and duct of Santorini morphology. Gut Liver 2018; 12: 208–13. 29212312 10.5009/gnl17170 PMC5832346

[8] Suzuki A , Ishii S , Fujisawa T *et al*. Efficacy and safety of peroral pancreatoscopy through the fistula created by endoscopic ultrasound‐guided pancreaticogastrostomy. Pancreas 2022; 51: 228–33. 35584379 10.1097/MPA.0000000000002003

[9] Sanada Y , Yoshida K , Itoh H , Kunita S , Jinushi K , Matsuura H . Groove pancreatitis associated with true pancreatic cyst. J Hepatobiliary Pancreat Surg 2007; 14: 401–9. 17653641 10.1007/s00534-006-1180-7

[10] Bhutani MS . Interventional endoscopic ultrasonography: State of the art at the new millenium. Endoscopy 2000; 32: 62–71. 10691275 10.1055/s-2000-139

